# Supplementary material for: Structures illustrate step-by-step mitochondrial transcription initiation
Source: Nature. 2023 Oct 11;622(7984):872–9. doi: 10.1038/s41586-023-06643-y (PMC10600007; doi:10.1038/s41586-023-06643-y)
Supplement: Supplementary file 1 — Supplementary Figs. 1–3 and legends for Supplementary Videos 1 and 2. [file 41586_2023_6643_MOESM1_ESM.pdf]

nature portfolio

https://doi.org/10.1038/s41586-023-06643-y

Supplementary information

Structures illustrate step-by-step mitochondrial transcription initiation

In the format provided by the authors and unedited

Supplementary Information for:

Structures illustrate step-by-step mitochondrial transcription initiation

Quinten Goovaerts[1,2,#], Jiayu Shen[3,#], Brent De Wijngaert[1,2], Urmimala Basu[3], Smita S. Patel[3,*] and Kalyan Das[1,2,*]

[1]Laboratory of Virology and Chemotherapy, Rega Institute for Medical Research, KU Leuven, 3000 Leuven, Belgium

[2]Department of Microbiology, Immunology and Transplantation, KU Leuven, 3000 Leuven, Belgium

[3]Department of Biochemistry and Molecular Biology, Robert Wood Johnson Medical School, Rutgers University, Piscataway, NJ 08854, USA

[#]Contributed equally

*Corresponding authors: kalyan.das@kuleuven.be (KD), patelss@rwjms.rutgers.edu (SSP)

Table of content:

Expanded legends for Supplementary Videos 1 and 2…..………………………page 3-4

Supplementary Figure 1 (Uncropped gels)………..……………………………...page 5

Supplementary Figure 2 (Cryo-EM image processing flowcharts)……………..page 6-11

Supplementary Figure 3 (3D FSC plots for cryo-EM maps)…………..………...page 12-18

Legend Supplementary Video 1. Structural changes in the transcription bubble after each nucleotide addition from PmIC to IC8 and EC. The y-mtRNAP is in blue, MTF1 in yellow, non-template (NT) DNA in cyan, template DNA in pink, incoming NTPs in blue, and the incorporated RNA in green C-atom representation complementing the red template; the template position -1 is in green. In the movie, each end-state is shown in color, and the start-state is gray. The y-mtRNAP and MTF1 are faded for clear visualization of the transcription bubble.

Step 1. PmIC → IC2 transition: (i) the transcription bubble engages two GTP molecules at the active site to initiate *de novo* RNA synthesis, (ii) the promoter melting is accompanied by an additional 60° bending of the downstream DNA, (iii) the template single-strand part assumes a "U" shape, and (iii) the non-template (NT) strand scrunches into a loop.

Step 2. IC2 → IC3 transition: (i) the third nucleotide (an UTPαS) binds at the N site and is poised for catalytic incorporation, (ii) the "U" shape template starts to bulge as the RNA:DNA duplex pushes the single strand template region of -4 to -1 nucleotides, and (iii) the NT loop scrunches further.

Step 3. IC3 → IC4 transition: (i) the 4th nucleotide incorporation to the RNA strand, (ii) the template bulge expands in size, and the -1 template base (in green) that is stacked with the RNA:DNA in IC2/IC3 unstacks, and (iii) the scrunched NT loop relaxes. The single-strand parts of the template and non-template in the transcription bubble are less ordered in IC4.

Step 4. IC4 → IC5 transition: (i) the 5-mer RNA is formed, (ii) surprisingly, the NT strand switches its position and its +1 to +6 nucleotide bases stack as a "staircase-like" structure, and (iii) the unpaired template +6 base is ready to enter the polymerase cleft to base pair with the next NTP.

Step 5. IC5 → IC6 transition: (i) the 6-mer RNA is formed and (ii) little change in the NT strand conformation.

Step 6. IC6 → IC7 transition: (i) the 7-mer RNA is formed, (ii) the NT stack gets larger with the stacking of +7 base, and (iii) the template bulges further at position -1.

Step 7. IC7 → IC8 transition: (i) the RNA is elongated to 8-mer and the 3'-end of the RNA:DNA translocates from the N-site to the P-site, shifting the hybrid towards the upstream promoter region by 2 nucleotides, (ii) the NT staircase inflates to push MTF1 away from the thumb, and (iii) the template scrunches significantly at the -1 position and consequently repositions the -1 to -3 bases facing toward the NT strand for bubble collapse.

Step 8. The IC8 → EC transition is modeled: The IC8 structure with the mismatch bubble (-4 to +2) promoter captures an intermediate state prior to bubble collapse. The y-mtRNAP EC structure was modeled from h-mtRNAP EC structure (PDB ID. 4BOC). Multiple conformational changes upon 8-mer synthesis would collectively trigger the initiation of bubble collapse, MTF1

dissociation, and upstream promoter release. The released upstream DNA segment undergoes a large 120° DNA unbending rotation to a new location occupied earlier by the C-terminal domain of MTF1.

In the end, all eight transition steps are shown as a single uninterpreted clip; starting at timestamp 2:35

Legend Supplementary Video 2. Structural changes in y-mtRNAP and MTF1 after each nucleotide addition from PmIC to IC8 and EC.

The nucleic acid parts are faded for a clearer view of proteins.

Step 1. PmIC → IC2 transition: MTF1 slides over y-mtRNAP to close the polymerase cleft and the downstream cleft, which accommodates the downstream DNA to support a fully expanded transcriptional bubble and *de novo* RNA synthesis.

Steps 2 & 3. IC2→ IC3→ IC4 transitions: the polymerase cleft opens gradually with each nucleotide addition to accommodate the expanding transcription bubble. In IC4, the relative positioning of MTF1 and y-mtRNAP fall back to their respective positions observed in PmIC.

Steps 4 – 6. IC4→ IC5→ IC6→ IC7 transitions: MTF1 moves in a lateral direction with respect to y-mtRNAP, which is ~95º from its closing/opening movements observed in the PmIC to IC4 transitions. This direction of MTF1 movement appears to be along a path that helps release MTF1.

Step 7. IC7 → IC8 transition: C-tail moves out of the polymerase cleft, and MTF1 and thumb dissociate and move away from each other.

Step 8. IC8 → EC transition: the MTF1 dissociates from y-mtRNAP and the promoter DNA. The MTF1-hairpin switches role from holding the C-terminal domain of MTF1 in IC states to accommodate the upstream DNA in EC.

In the end, all eight transition steps are shown as a single uninterpreted clip; starting at timestamp 2:33

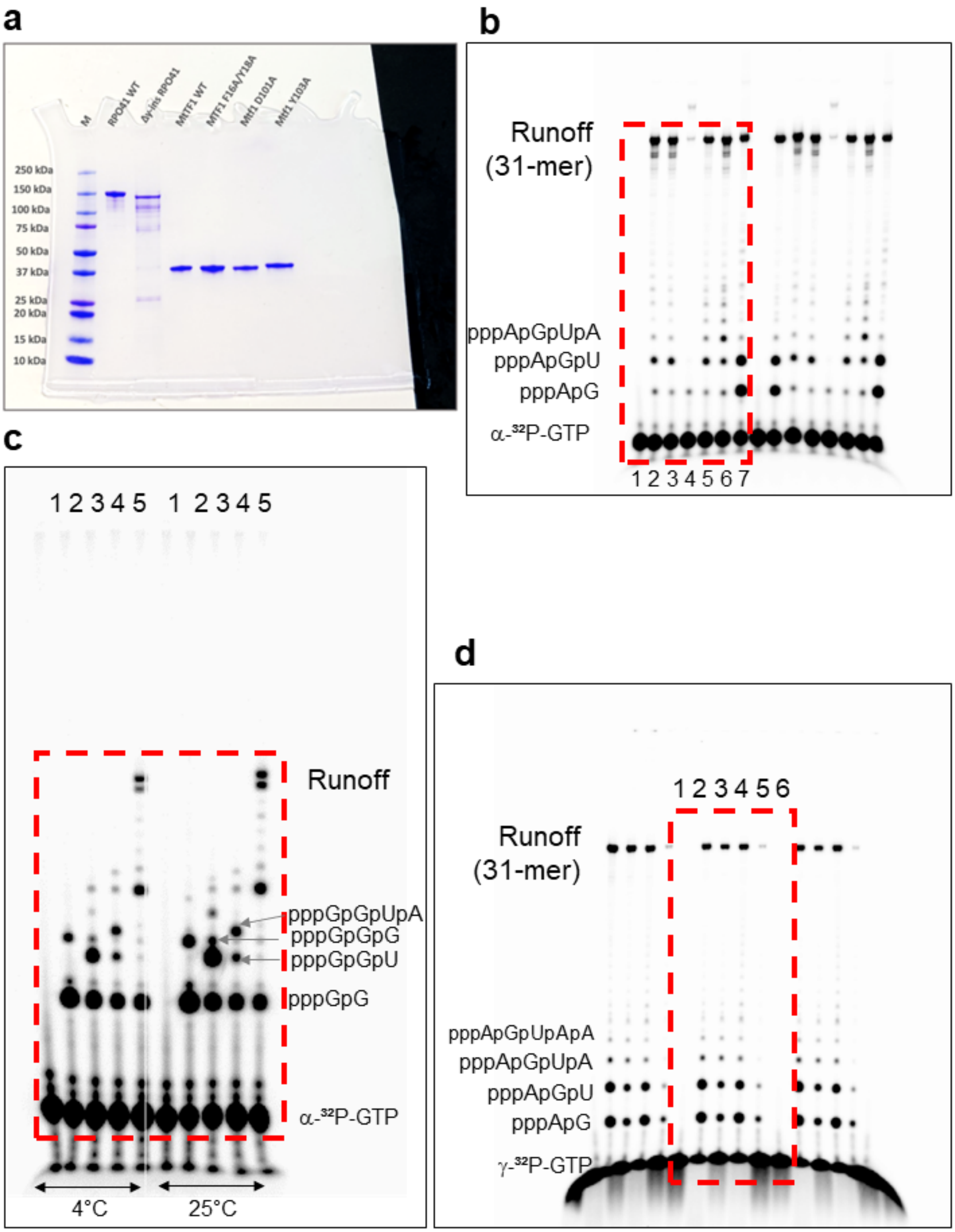

Supplementary Fig 1. Gel source images. a. Uncropped SDS-PAGE protein gel of different y-mtRNAP and MTF1 proteins. Purified wild-type and mutant proteins (y-mtRNAP and MTF1) are used in transcription runoff assays. **b.** Uncropped gel of Figure 2f. **c.** Uncropped gel of Extended Data figure 1b. **d.** Uncropped gel of Extended Data Figure 1c.

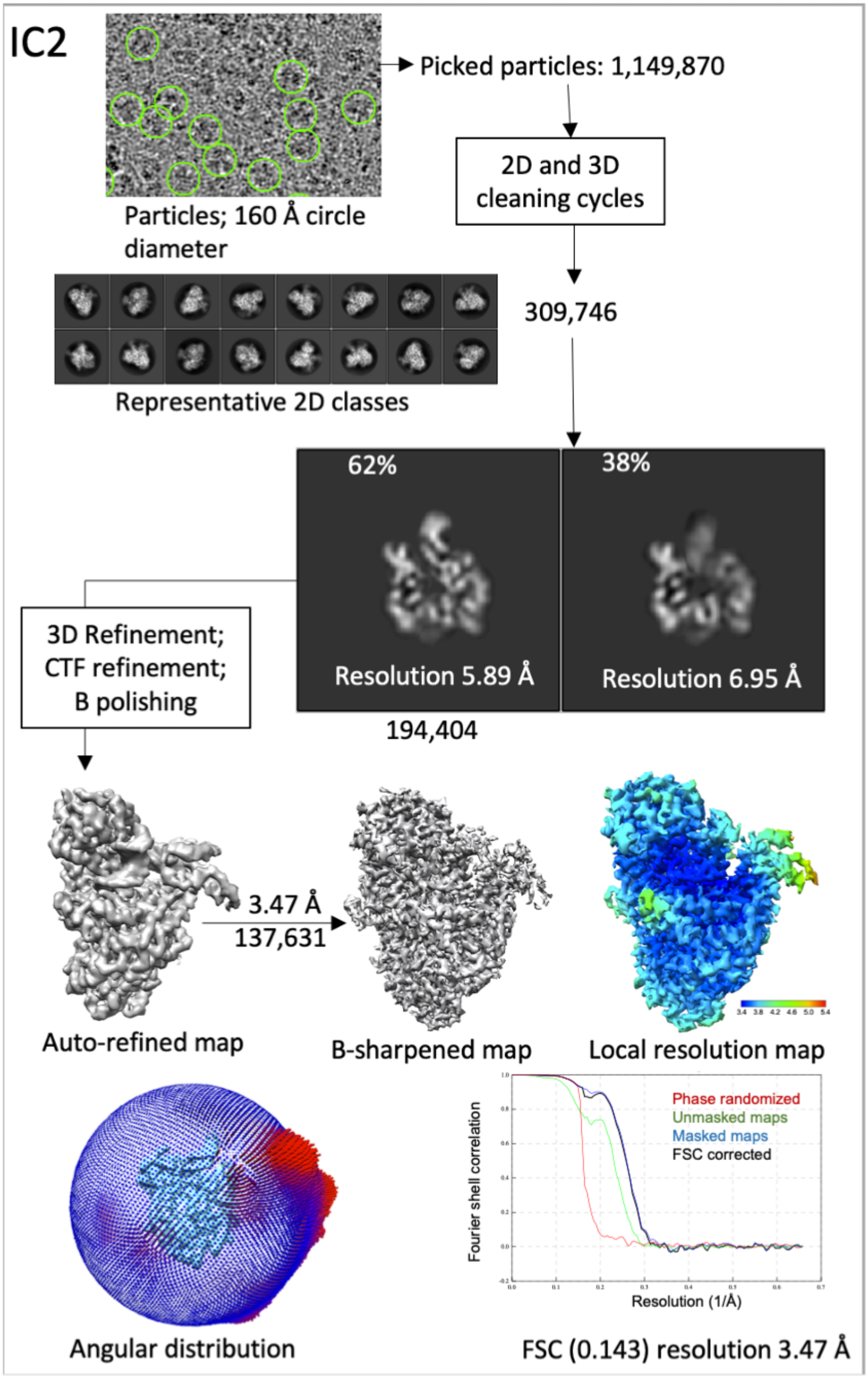

Supplementary Fig 2a. Cryo-EM image processing flowcharts summarizing the data processing and the density map quality, particle distribution and resolution of IC2.

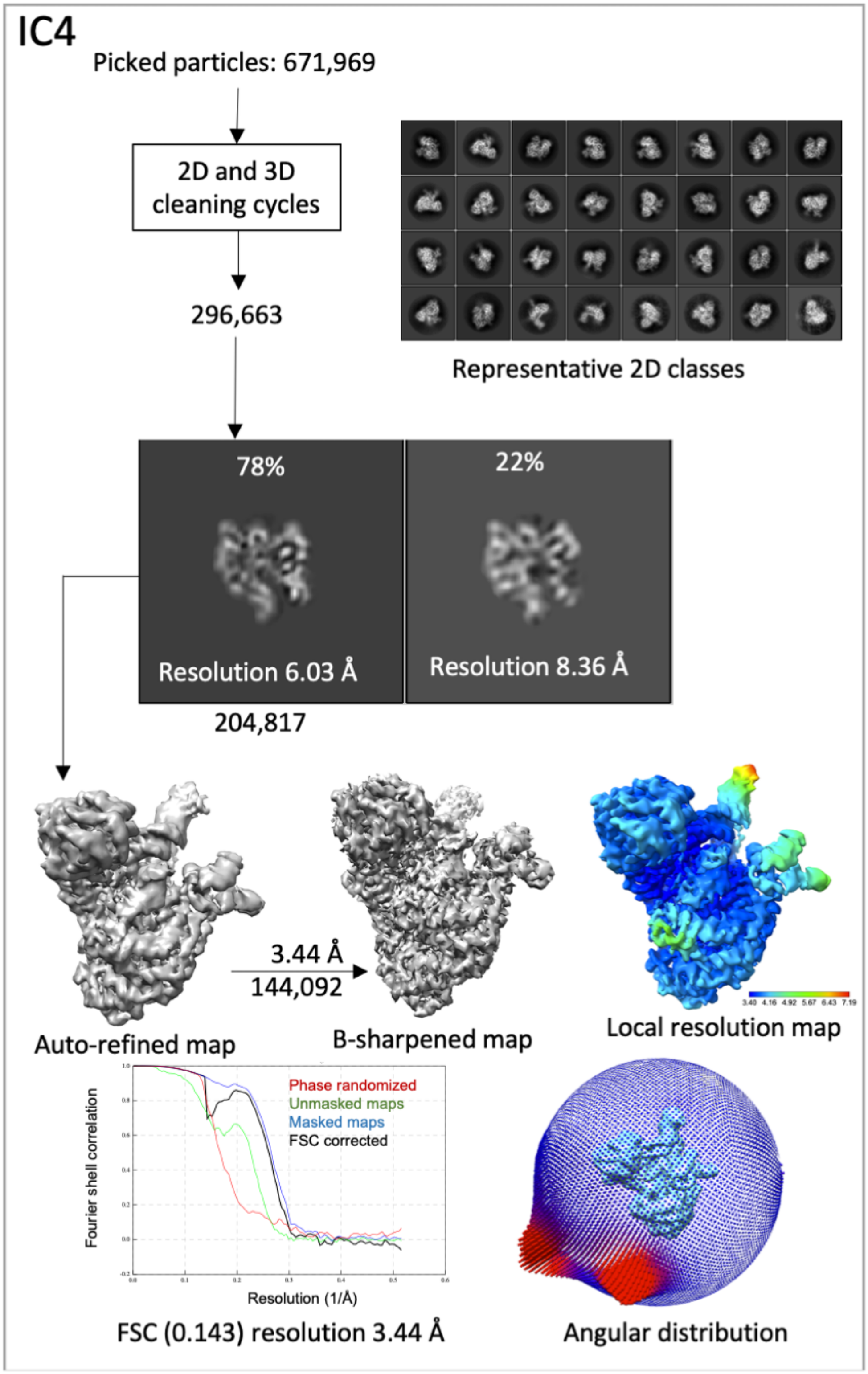

Supplementary Fig 2b. Cryo-EM image processing flowcharts summarizing the data processing and the density map quality, particle distribution and resolution of IC4.

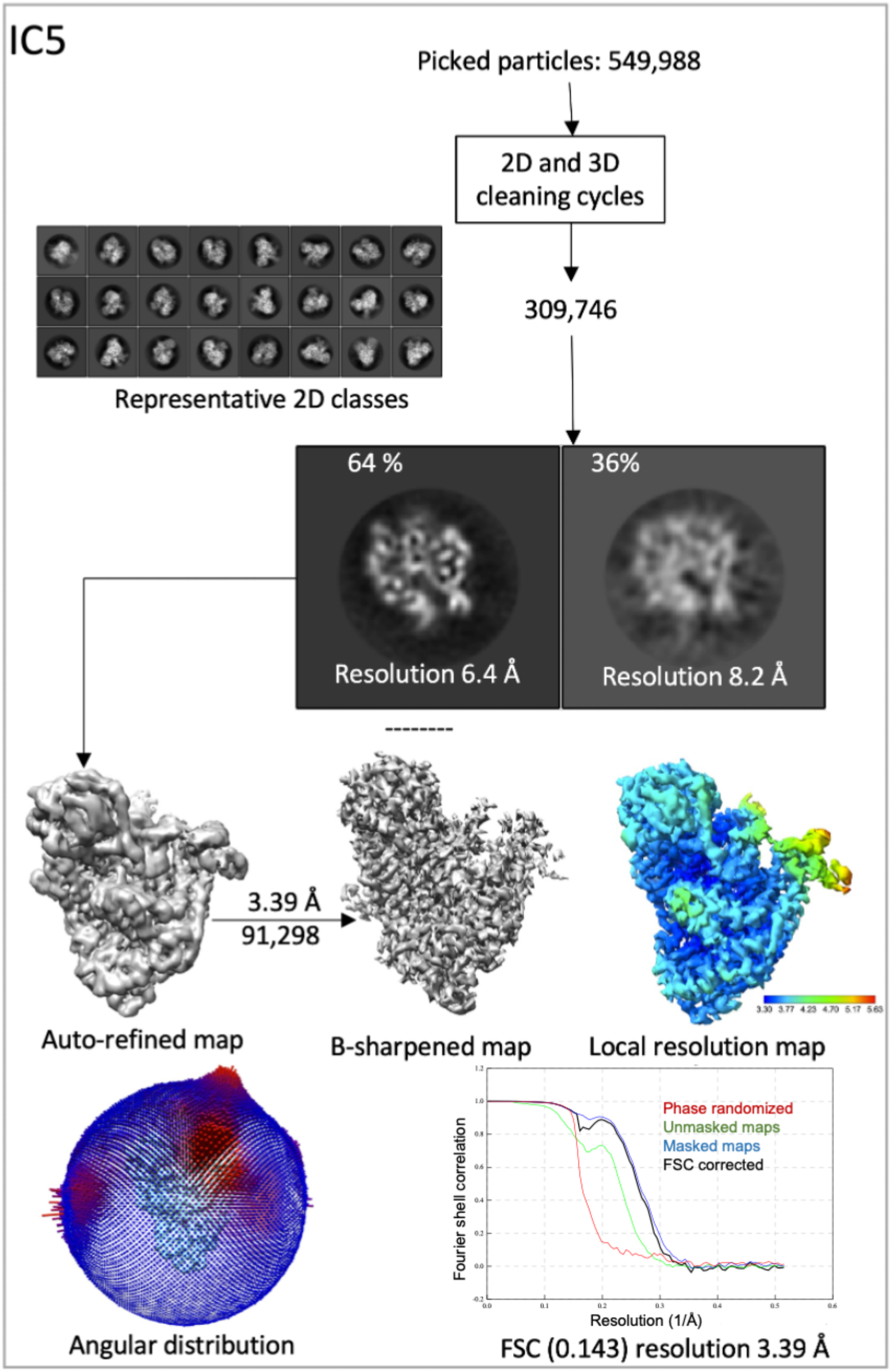

Supplementary Fig 2c. Cryo-EM image processing flowcharts summarizing the data processing and the density map quality, particle distribution and resolution of IC5.

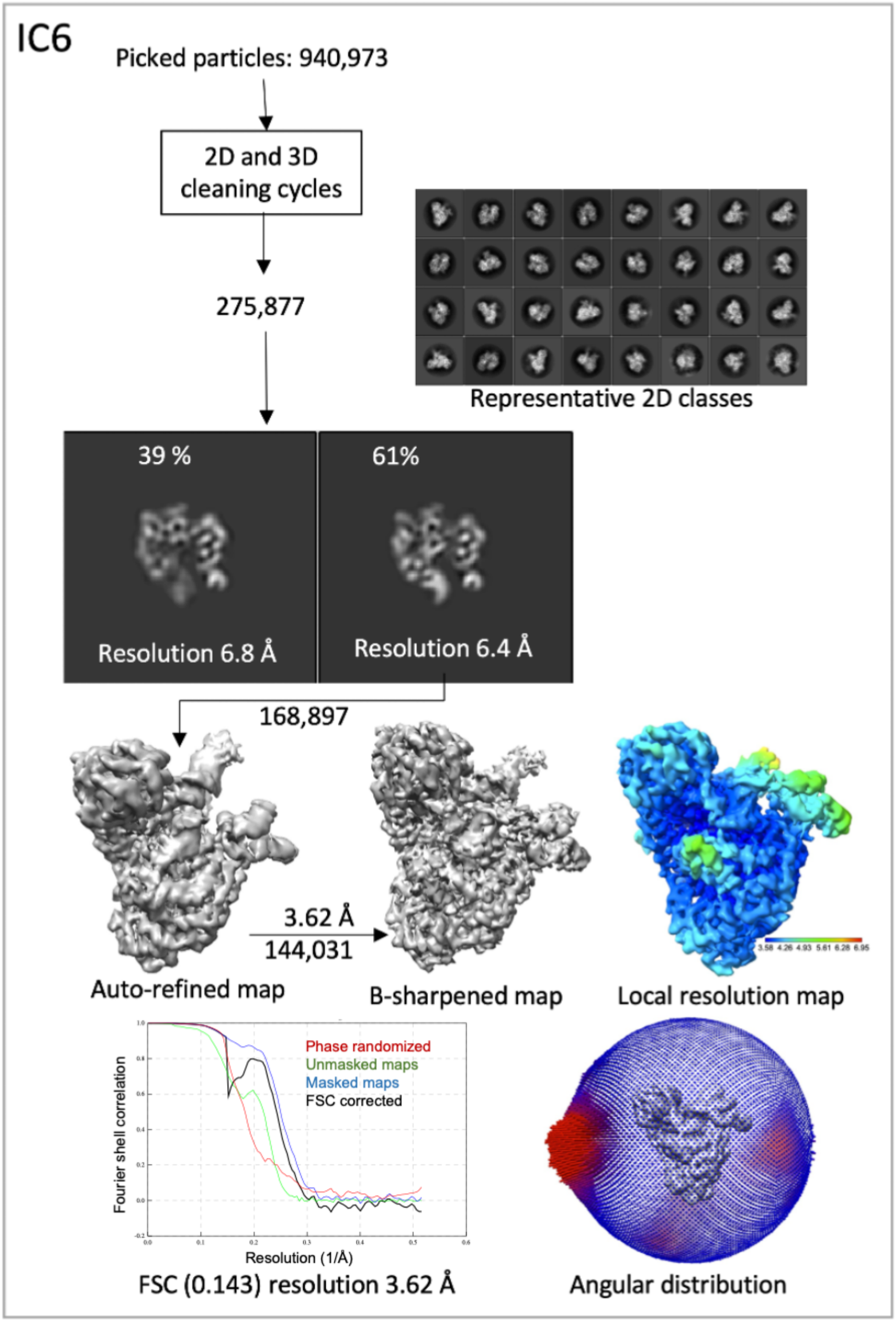

Supplementary Fig 2d. Cryo-EM image processing flowcharts summarizing the data processing and the density map quality, particle distribution and resolution of IC6.

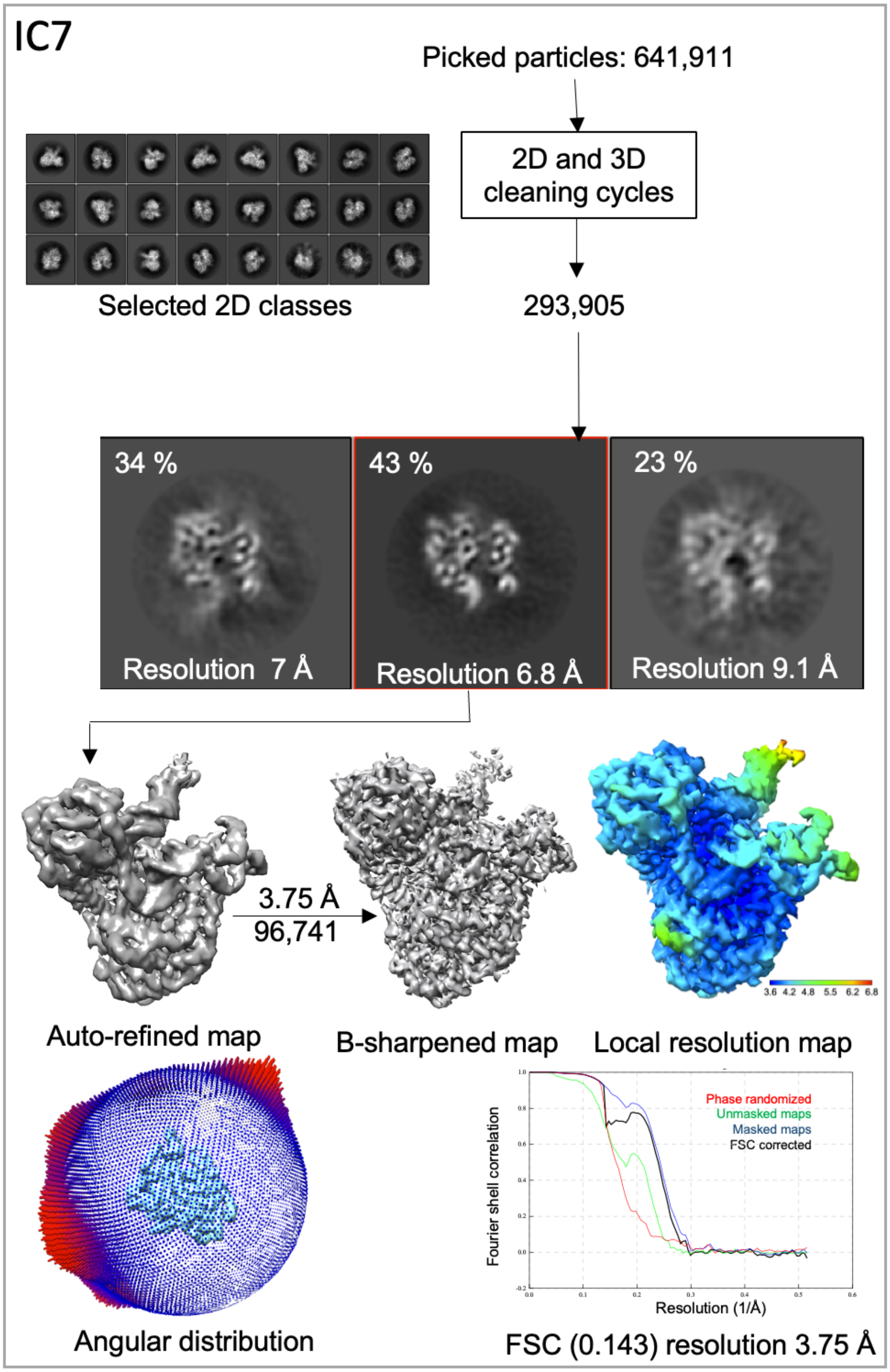

Supplementary Fig 2e. Cryo-EM image processing flowcharts summarizing the data processing and the density map quality, particle distribution and resolution of IC7.

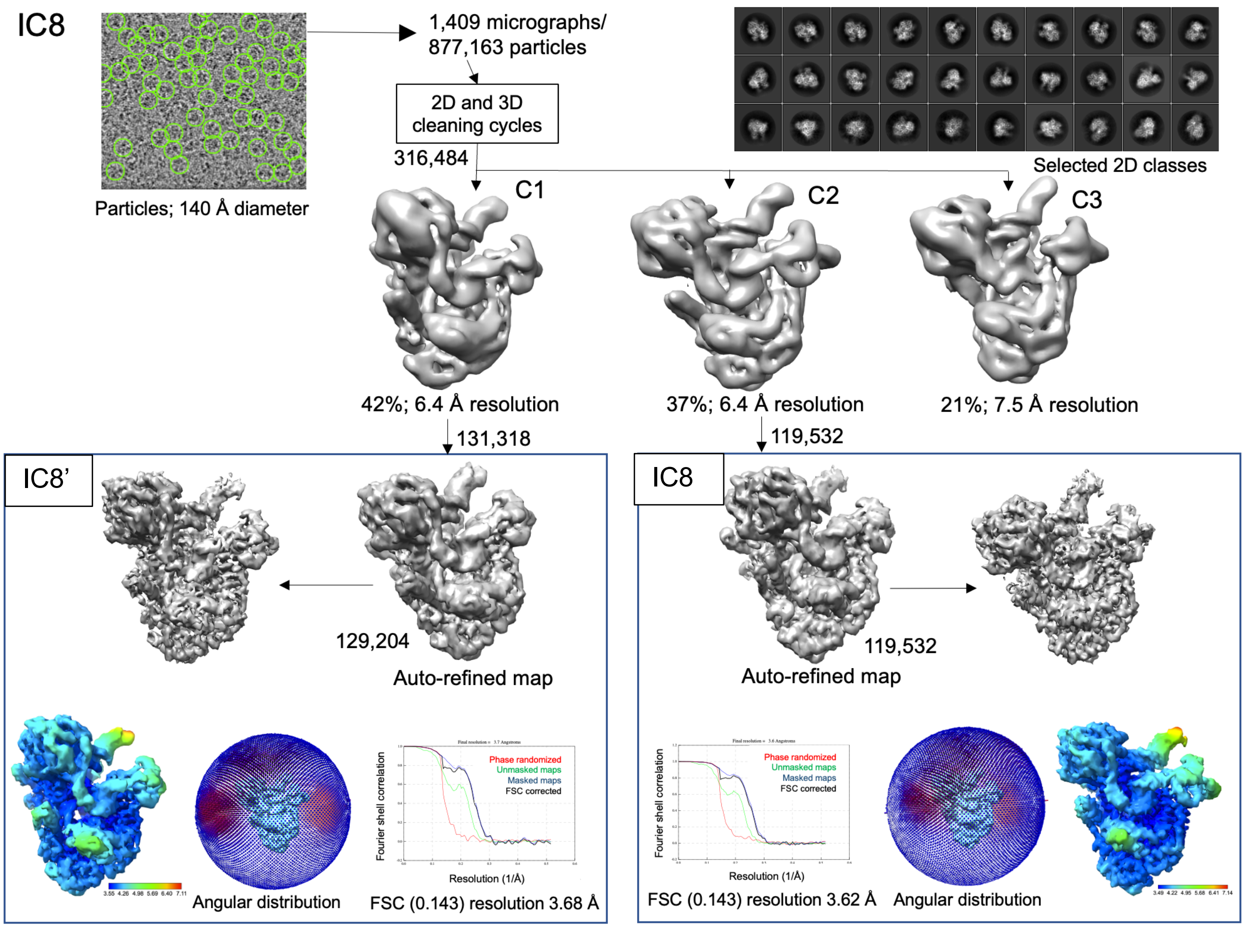

Supplementary Fig 2f. Cryo-EM image processing flowcharts summarizing the data processing and the density map quality, particle distribution and resolution of IC8 complexes. Two distinct populations of IC8 were obtained; however, no significant structural differences were observed between the two except the thumb and MTF1 are further apart by 5 and 3 Å (Extended Data Fig. 7d) in the left structure compared to the right one with resolution of 3.62 Å; the 3.62 Å resolution structure is used as the IC8 structure.

a <u>IC2</u>

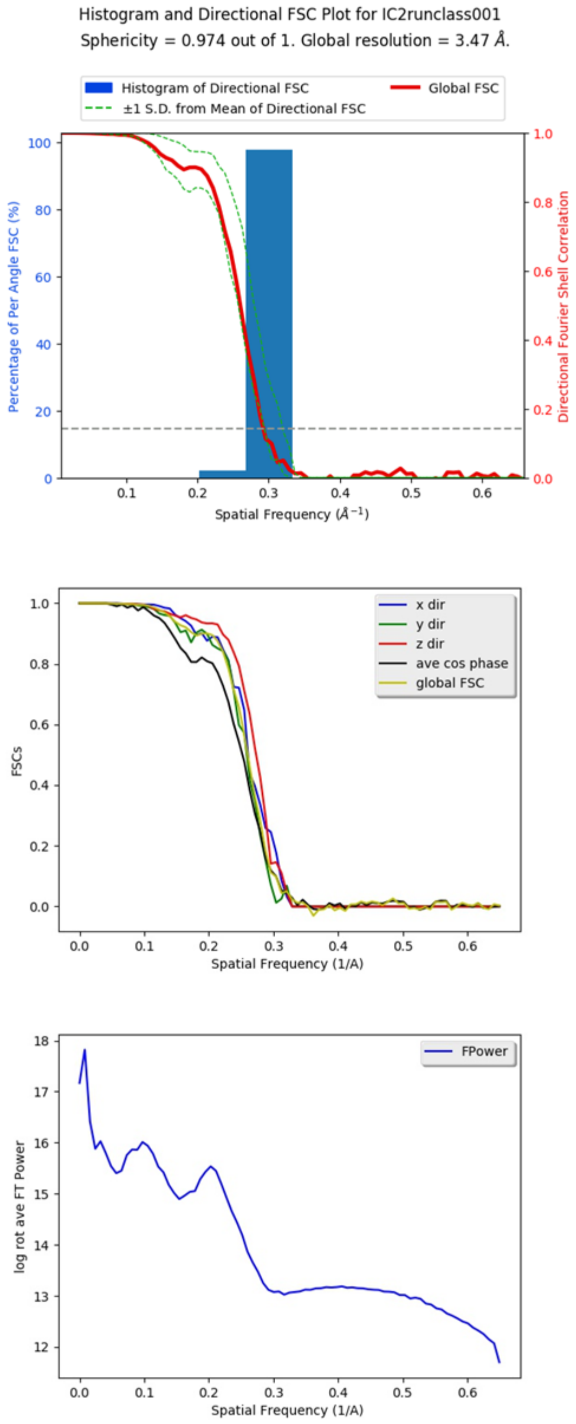

Supplementary Fig 3a. 3D FSC plots for the cryo-EM map of IC2 calculated with https://3dfsc.salk.edu/.

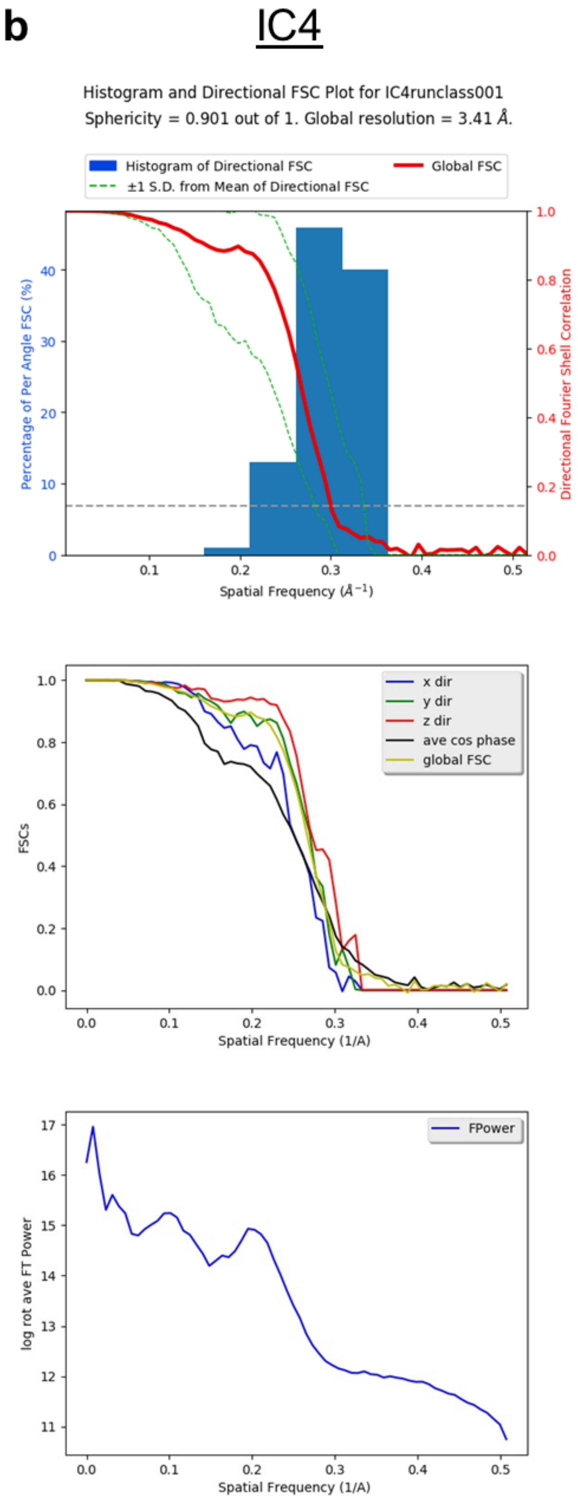

Supplementary Fig 3b. 3D FSC plots for the cryo-EM map of IC4 calculated with https://3dfsc.salk.edu/.

c IC5

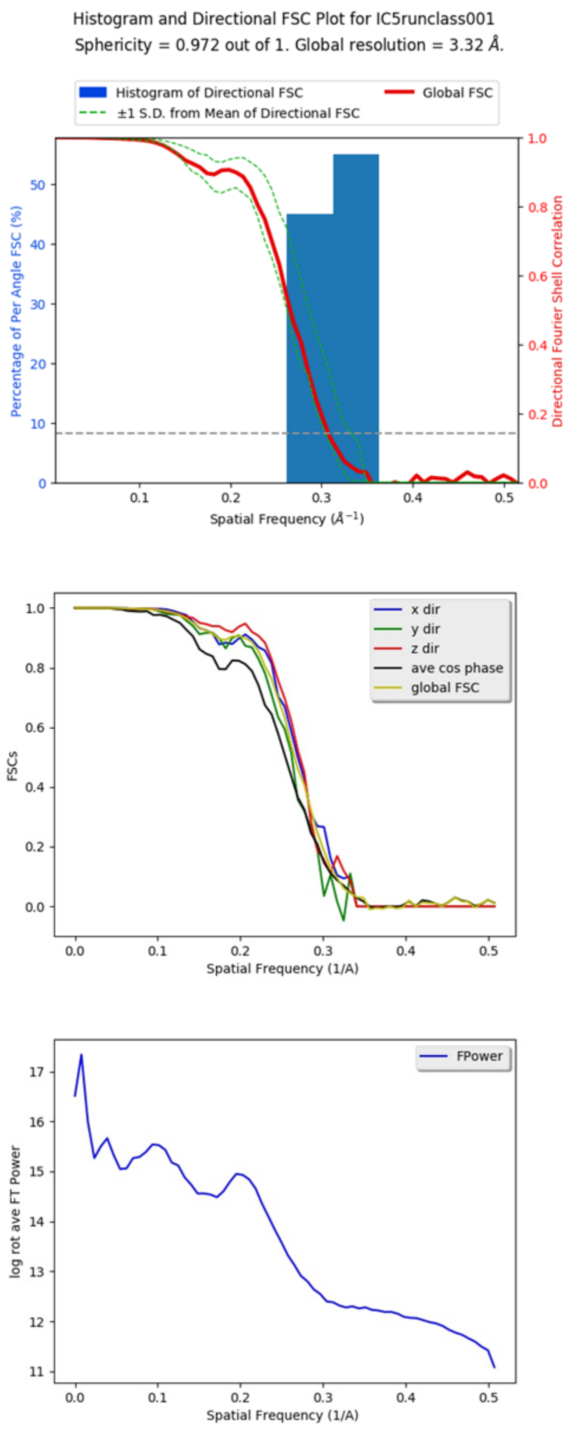

Supplementary Fig 3c. 3D FSC plots for the cryo-EM map of IC5 calculated with https://3dfsc.salk.edu/.

d IC6

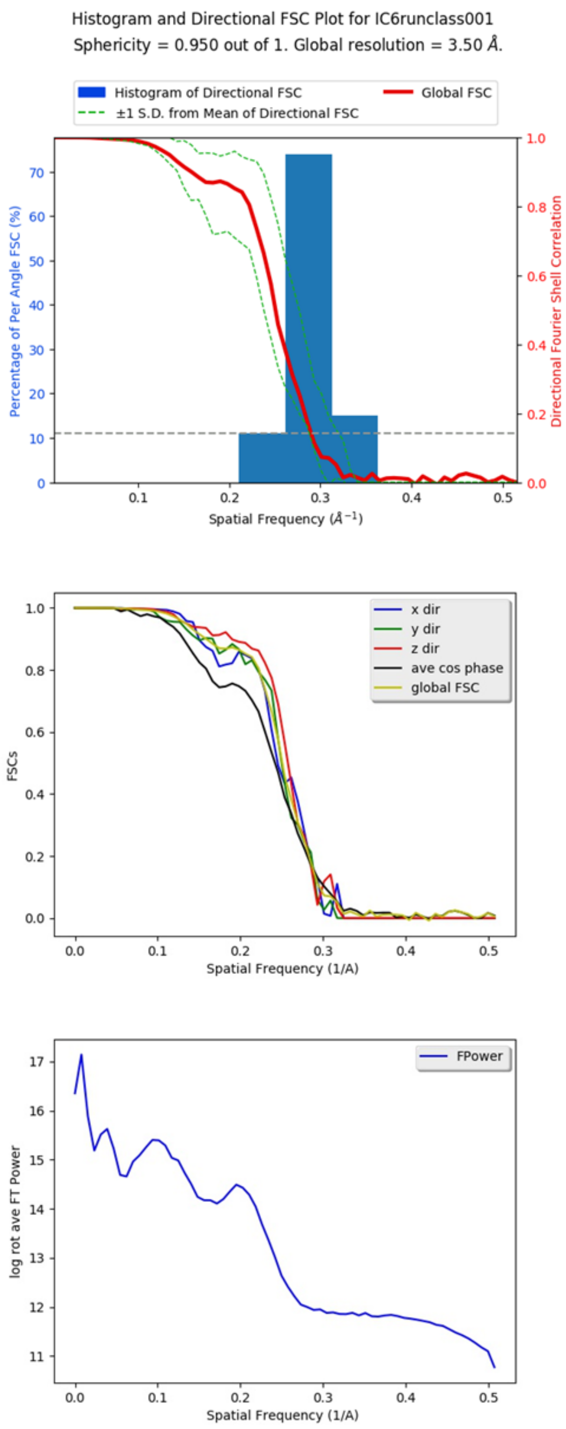

Supplementary Fig 3d. 3D FSC plots for the cryo-EM map of IC6 calculated with https://3dfsc.salk.edu/.

e

IC7

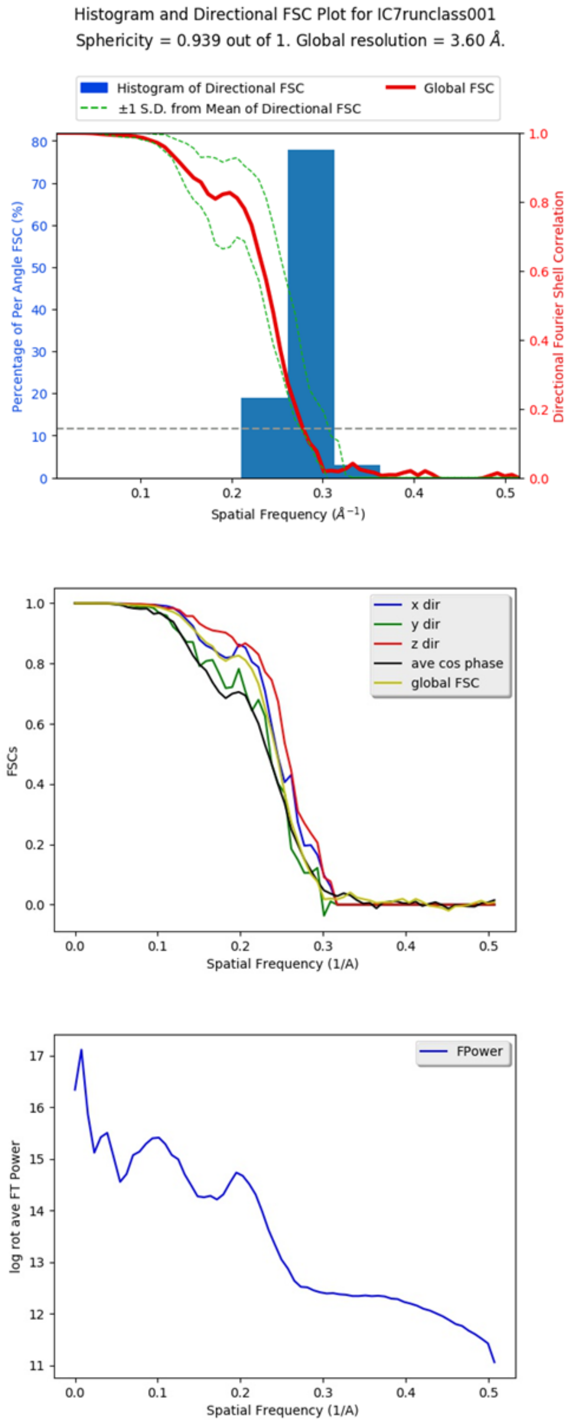

Supplementary Fig 3e. 3D FSC plots for the cryo-EM map of IC7 calculated with https://3dfsc.salk.edu/.

f IC8

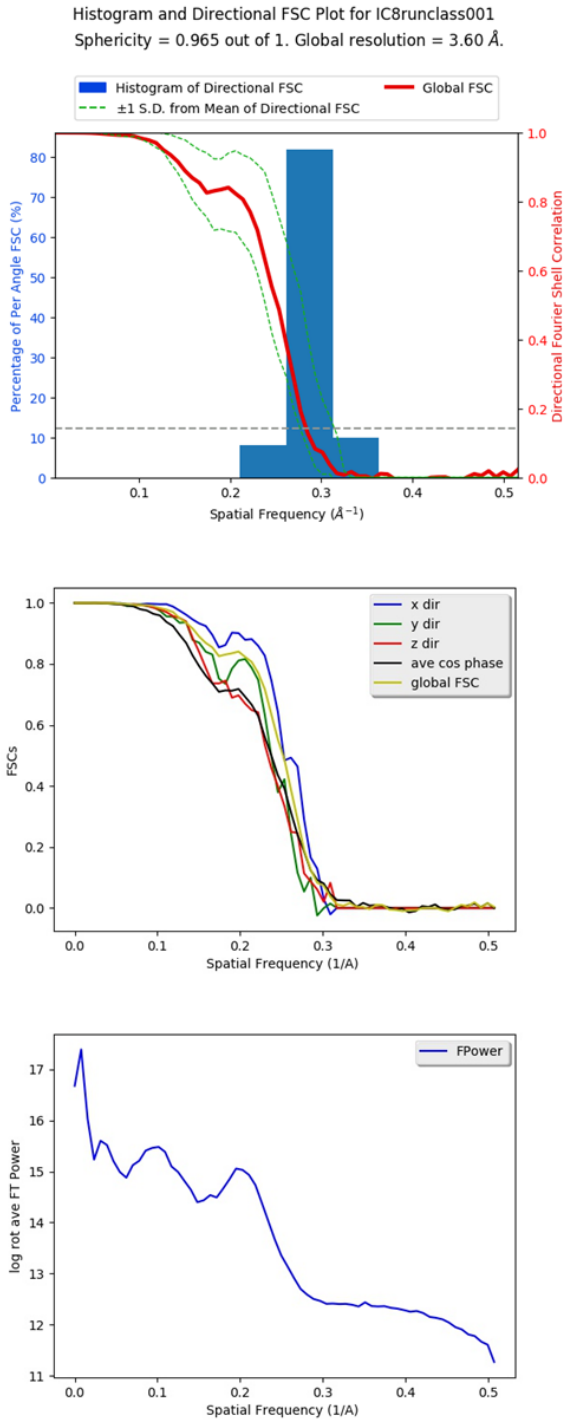

Supplementary Fig 3f. 3D FSC plots for the cryo-EM map of IC8 calculated with https://3dfsc.salk.edu/.

g IC8’

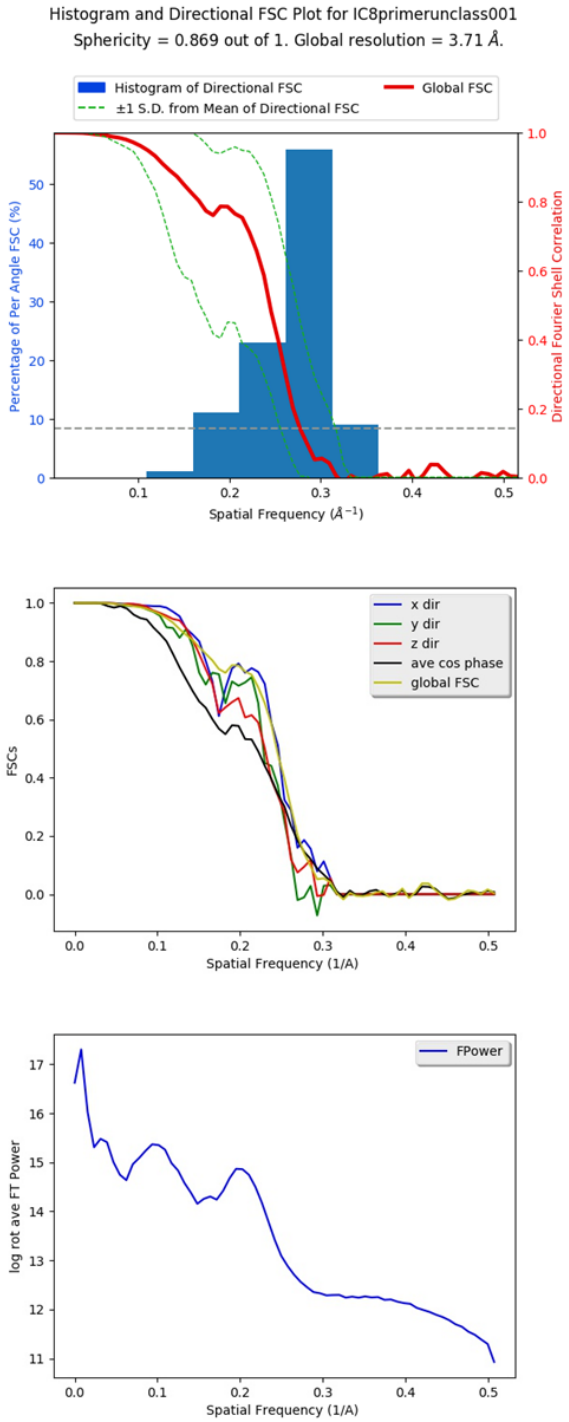

Supplementary Fig 3g. 3D FSC plots for the cryo-EM map of IC8’ calculated with https://3dfsc.salk.edu/.
