# Supplementary material for: Structures illustrate step-by-step mitochondrial transcription initiation
Source: Nature. 2023 Oct 11;622(7984):872–9. doi: 10.1038/s41586-023-06643-y (PMC10600007; doi:10.1038/s41586-023-06643-y)
Supplement: Supplementary file 2 — Reporting Summary [file 41586_2023_6643_MOESM2_ESM.pdf]

nature portfolio

Corresponding author(s): Kalyan Das

Last updated by author(s): Aug 16, 2023

Reporting Summary

Nature Portfolio wishes to improve the reproducibility of the work that we publish. This form provides structure for consistency and transparency in reporting. For further information on Nature Portfolio policies, see our Editorial Policies and the Editorial Policy Checklist.

Statistics

For all statistical analyses, confirm that the following items are present in the figure legend, table legend, main text, or Methods section.

n/a	Confirmed	
☐	☒	The exact sample size (*n*) for each experimental group/condition, given as a discrete number and unit of measurement
☐	☒	A statement on whether measurements were taken from distinct samples or whether the same sample was measured repeatedly
☒	☐	The statistical test(s) used AND whether they are one- or two-sided<br>*Only common tests should be described solely by name; describe more complex techniques in the Methods section.*
☒	☐	A description of all covariates tested
☒	☐	A description of any assumptions or corrections, such as tests of normality and adjustment for multiple comparisons
☐	☒	A full description of the statistical parameters including central tendency (e.g. means) or other basic estimates (e.g. regression coefficient) AND variation (e.g. standard deviation) or associated estimates of uncertainty (e.g. confidence intervals)
☒	☐	For null hypothesis testing, the test statistic (e.g. F, t, r) with confidence intervals, effect sizes, degrees of freedom and P value noted<br>*Give P values as exact values whenever suitable.*
☒	☐	For Bayesian analysis, information on the choice of priors and Markov chain Monte Carlo settings
☒	☐	For hierarchical and complex designs, identification of the appropriate level for tests and full reporting of outcomes
☒	☐	Estimates of effect sizes (e.g. Cohen's *d*, Pearson's *r*), indicating how they were calculated

Our web collection on statistics for biologists contains articles on many of the points above.

Software and code

Policy information about availability of computer code

Data collection	EPU 2.9.0 (for single-particle cryo-EM data collection on Glacios)
Data analysis	Relion 3.1, CTFFIND-4 and ResMap 1.1.5 for data-processing; Coot 0.9.4 and Phenix 1.19.2-4158 for model building and structure refinement; PyMol 2.4.1, Chimera 1.15rc, ChimeraX 1.2.5 to generate structural figures and movies. ImageQuant version 7 for visualization, quantification and analysis of transcription assays. GraphPad Prism 9.5.1 for visualization and plot presentation of transcription assays. Dynamics software version 7.10.0.23 to confirm hydrodynamic radius and polydispersity. 3DFSC webserver for 3D-FSC cryo-EM plots.

For manuscripts utilizing custom algorithms or software that are central to the research but not yet described in published literature, software must be made available to editors and reviewers. We strongly encourage code deposition in a community repository (e.g. GitHub). See the Nature Portfolio guidelines for submitting code & software for further information.

Data

Policy information about availability of data

All manuscripts must include a data availability statement. This statement should provide the following information, where applicable:

- Accession codes, unique identifiers, or web links for publicly available datasets
- A description of any restrictions on data availability
- For clinical datasets or third party data, please ensure that the statement adheres to our policy

The coordinates and cryo-EM density maps for IC2, IC4, IC5, IC6, IC7, IC8 and IC8' have been deposited under PDB and EMDataBank accession codes 8AP1, 8ATT,

8ATV, 8ATW, 8C5S, 8C5U and 8Q63, and EMD-15556, EMD-15662, EMD-15664, EMD-15665, EMD-16442, EMD-16443 and EMD-18183, respectively. For model building, the following models from PDB were used: 6YMV and 6YMW. AlphaFold accession number P13433 was used for γ-ins region model building. All other related data generated and/or analyzed during this research, such as raw cryo-EM images, are available from the corresponding authors upon reasonable request. Source data and raw gels are also available.

Research involving human participants, their data, or biological material

Policy information about studies with human participants or human data. See also policy information about sex, gender (identity/presentation), and sexual orientation and race, ethnicity and racism.

Reporting on sex and gender	N/A
Reporting on race, ethnicity, or other socially relevant groupings	N/A
Population characteristics	N/A
Recruitment	N/A
Ethics oversight	N/A

Note that full information on the approval of the study protocol must also be provided in the manuscript.

Field-specific reporting

Please select the one below that is the best fit for your research. If you are not sure, read the appropriate sections before making your selection.

☒ Life sciences ☐ Behavioural & social sciences ☐ Ecological, evolutionary & environmental sciences

For a reference copy of the document with all sections, see nature.com/documents/nr-reporting-summary-flat.pdf

Life sciences study design

All studies must disclose on these points even when the disclosure is negative.

Sample size	No statistical methods were used for sample size calculations. Six cryo-EM data sets were collected on the yeast mitochondrial RNA polymerase transcription initiation complex and all data sets yielded sufficient amount of good particles after classification to obtain high-resolution density maps. Biochemical and functional assays have been repeated 2 to 3 times independently and showed to have excellent reproducibility.
Data exclusions	No data was excluded for this study. Only poor particles were discarded during the cryo-EM data processing as it is a vital part of the workflow.
Replication	All biochemical and functional assays have been repeated 2 to 3 times independently and indicated reproducibility. Cryo-EM images were reproducibly obtained on different days, collections and with different protein purifications.
Randomization	Randomization was not performed as experimental groups are not needed for this work and thus it is not applicable for this current study.
Blinding	No experimental groups or randomization is required for this work, hence blinding is not applicable to this study.

Reporting for specific materials, systems and methods

We require information from authors about some types of materials, experimental systems and methods used in many studies. Here, indicate whether each material, system or method listed is relevant to your study. If you are not sure if a list item applies to your research, read the appropriate section before selecting a response.

Materials & experimental systems

n/a	Involved in the study
☒	☐ Antibodies
☒	☐ Eukaryotic cell lines
☒	☐ Palaeontology and archaeology
☒	☐ Animals and other organisms
☒	☐ Clinical data
☒	☐ Dual use research of concern
☒	☐ Plants

Methods

n/a	Involved in the study
☒	☐ ChIP-seq
☒	☐ Flow cytometry
☒	☐ MRI-based neuroimaging
